# Automation of Measurements for Personalized Medical Appliances by Means of CAD Software—Application in Robin Sequence Orthodontic Appliances

**DOI:** 10.3390/bioengineering9120773

**Published:** 2022-12-06

**Authors:** Maite Aretxabaleta, Ariadne Roehler, Christian F. Poets, Alexander B. Xepapadeas, Bernd Koos, Christina Weise

**Affiliations:** 1 Department of Orthodontics in the University Centre of Dentistry, Oral Medicine and Maxillofacial Surgery, University Hospital Tübingen, Osianderstr. 2-8, 72076 Tübingen, Germany.; 2 Medical Materials Science and Technology, University Hospital Tübingen, Osianderstr. 2-8, 72076 Tübingen, Germany; 3 Department of Neonatology, University Hospital Tübingen, Calwerstr. 7, 72076 Tübingen, Germany

**Keywords:** human error reduction, Rhino 7, Grasshopper, anatomical shapes, digitalization, Tübingen Palatal Plate, scanning, reproducibility, craniofacial anomalies, individualized medicine

## Abstract

Measuring the dimensions of personalized devices can provide relevant information for the production of future such devices used in various medical specialties. Difficulties with standardizing such measurement and obtaining high accuracy, alongside cost-intensive measuring methodologies, has dampened interest in this practice. This study presents a methodology for automatized measurements of personalized medical appliances of variable shape, in this case an orthodontic appliance known as Tübingen Palatal Plate (TPP). Parameters such as length, width and angle could help to standardize and improve its future use. A semi-automatic and custom-made program, based on Rhinoceros 7 and Grasshopper, was developed to measure the device (via an extraoral scanner digital file). The program has a user interface that allows the import of the desired part, where the user is able to select the necessary landmarks. From there, the program is able to process the digital file, calculate the necessary dimensions automatically and directly export all measurements into a document for further processing. In this way, a solution for reducing the time for measuring multiple dimensions and parts while reducing human error can be achieved.

## 1. Introduction

For the successful treatment of certain conditions, medicine must rely on patient-specific or personalized medical devices. These individualized devices provide support in various indications, from implants (e.g., as used in craniomaxillofacial surgery [1]) to prosthetic and orthotic appliances (e.g., spinal braces and forearm static fixation devices) [2]. A perfect adaptation of the device to the patient’s anatomy is essential, not solely for achieving good rehabilitative functioning, but also to avoid complications or side effects (e.g., blistering, ulcers, discomfort) [2]. These devices are produced based on the individual’s anatomical information and obtained by different imaging techniques, such as CT-scans for internal body imaging [1] or scanners for body surface imaging [2]. Based on these data, a personalized appliance may be manufactured. Given technological advances, CAD/CAM (Computer-Aided Design and Computer-Aided Manufacturing) technologies are securing their place in the market of personalized devices [3,4,5]. By means of different CAD software, patient-specific devices can be designed and manufactured through different CAM technologies, such as additive (AM) or subtractive manufacturing (SM). 

The analysis of such personalized devices is an interesting field of study to further improve treatment. The retrospective study of successfully and unsuccessfully implemented individualized medical appliances can lead to valuable information towards a higher degree of standardization and treatment quality, as well as their improvement and optimization. Although individualization of devices is still necessary for successful treatment outcome, a lack of minimum standardization or guidelines leads to multiple try and error fittings, that apart from being time and cost-consuming, directly affect both patient and clinical staff. Personalized devices are usually shaped along an anatomical structure, comprised of only or predominantly organic shapes, while lacking geometrical ones. These shapes pose a challenge for quantification and description, as such measurements are usually obtained manually, which is prone to human error [6]. 

Historically, measurements with rulers and callipers, as well as the use of photography, have been the gold standard. These followed a protocol for an acceptable reproduction of measurements, started by an agreement on and selection of reproducible landmarks by means of various experts in the field. In this way, the process could be validated for the reliability of experts selecting the same landmarks for measurements [7]. The associated workload and measuring times are not only cost-intensive, but also prone to measurement errors [8,9]. Moreover, certain shapes could not be measured manually due to complex structures that made the positioning of callipers impossible. All of these factors may contribute to a decreased interest in performing such studies. 

With the increasing abilities of scanners alongside CAD software, a higher degree of measurement automation and reduction in human error has become possible. This is the scenario of manufacturing accuracy and volume comparison, where special CAD software allows for a fast and automated measurement [10]. When it comes to dimensional measurement instead, literature on medical device measurements is scarce. This may be due to the high workload related to measuring common polygon-based 3D modelling with CAD software, where the user needs to have deep CAD software knowledge to measure all respective dimensions through the use of conventional commands. Moreover, polygon-based software is limited in its ability to reproduce curves smoothly, e.g., those seen in the human body. Meanwhile, NURBS (Non-Uniform Rational B-Splines)-based CAD modelling software such as Rhinoceros 3D (also known as Rhino) (Robert McNeel & Associates, Seattle, WA, USA) has raised interest in human body analysis [11] and medical device fabrication [12,13]. There have also been attempts at automated processing, measuring and modelling average 3D human body parts using software such as Rhino and Grasshopper (Robert McNeel & Associates) [11]. Grasshopper is an extension complementing the NURBS-based Rhino, used to generate programming algorithms. Therefore, this combination of programs has potential for analysing personalized devices and measurements in the medical field. Advantages of implementing an automated workflow for measuring medical devices include an avoidance of time-consuming and repetitive tasks [11], higher measurement accuracy and reductions in human error [6]. By using this combination, results in the Grasshopper algorithm can be displayed and controlled in the main Rhino interface for visualization in real time. Moreover, the program can deal with many parameters simultaneously, avoiding repetitive steps and reducing processing time [11]. Despite the implementation of Rhino in some medical fields, such as medical device design [12,13], literature on the topic is scarce. The authors are not aware that it has been adopted for a user interface-based automatization solution for non-geometrical shapes of personalized medical appliances.

**Figure 1:**
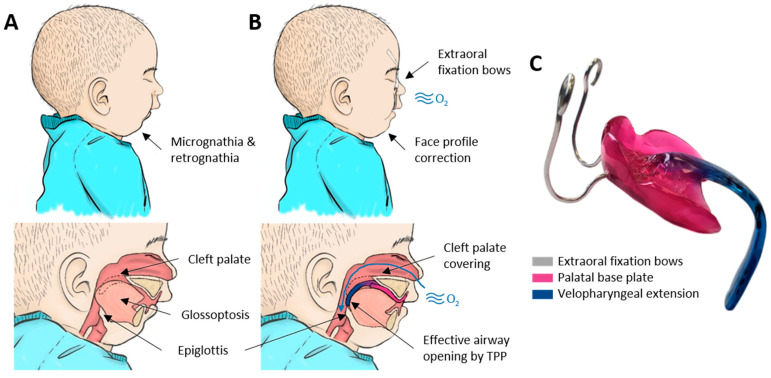
Robin sequence (RS) and its respective treatment by the Tübingen Palatal Plate (TPP). (**A**) RS is characterized as the combination of mandibular retrognathia, glossoptosis, and in the majority of the cases, a cleft palate, which all result in upper airway obstruction. (**B**) Correct placement of TPP leads to the subsequent airway opening and correct mandible positioning. (**C**) Example of an individualized TPP and its respective parts.

Among potential medical applications, such solution may have huge potential for the retrospective measurement of individualized orthodontic appliances as employed in the Robin sequence [14], i.e., the Tübingen Palatal Plate (TPP). This treatment is a renowned alternative to surgical methods for treating these patients [15,16,17,18,19,20,21,22,23,24,25,26,27,28,29] with favourable clinical results, which has been gaining international interest and has been adopted by different centres in recent years [30,31,32,33,34,35,36,37]. These orthodontic appliances are used in newborns and small infants with the objective of relieving upper airway obstruction (Figure 1A). The appliance consists of a base plate covering the palatal area and a velopharyngeal extension that ends just above the epiglottis (Figure 1C). This extension needs to be configurated perfectly so that it fits in a position where it is pushing the base of the tongue forward enough to open the airway, but without creating pressure marks on the soft tissue (Figure 1B). 

This medical device is based on an intraoral scan of the palatal area [38]. Unfortunately, this scan only provides the shape on which the maxillary is based, leaving the construction of the velopharyngeal extension to the extensive experience of the dental technician and clinician. The appliance is manufactured and the position of the velopharyngeal extension is checked via awake endoscopy [39,40]. This extension will be changed as often as necessary and re-checked using endoscopy until an optimal configuration is obtained. Moreover, patients receive between one and three TPPs, starting at birth, to adapt to their physiological growth. The fitting of the extension relies on a cumbersome and time-consuming process, given that no prior information of the necessary shape and length of the pharyngeal area exists [41]. The delicate situation of these patients prohibits further imaging techniques, such as CT scans or MRI. Moreover, this type of imaging technique will offer a “snapshot” and related measurements of the anatomical parts in a specific time and position (prone or supine). Therefore, personalized TPPs based on this kind of technique, such as the one proposed by Thurzo et al. [42], cannot be considered. As the appliance needs to promote the function alongside the movement of the tongue and pharyngeal area, a visualization method such as an endoscopy is still vital and will be the decisive step towards the acceptance of the appliance for treatment. To ensure the effectiveness of the extension, a polysomnography with a resultant mixed obstructive apnea index of less than three is also imperative [16]. Shape and dimensions of the extension are personalized for each patient, leading to a wide variety of extension dimensions (Figure 1). In this scenario, a retrospective study of the extension dimensions is desirable, as this can be related to clinical factors (such as head circumference), with the objective of standardizing the velopharyngeal extension design in the future and reducing fitting time as well as endoscopic procedures. Moreover, minimum dimensions need to be considered in the introduction of safety margins and quality control for the implementation of new materials and technologies for this treatment [41].

Therefore, this study aims to automatize measurements in personalized medical appliances with anatomic shapes, in this case the TPP, with the ultimate goal of presenting a feasible, fast, accurate and reliable solution to measure TPPs while reducing human interaction and, thus, human error as much as possible.

## 2. Materials and Methods

### 2.1. Sample Digitalization

For this proof-of-concept study, an extraoral scanner (Ceramill Map 400, Amann Girrbach AG, Knoblach, Austria; accuracy 20 µm) was employed to digitize TPP samples. An anonymized and successful TPP employed in a previous study was used as a base [41]. Prior to this, the appliance had been steam cleaned and dusted off with compressed air, followed by a homogeneous application of anti-glare spray (Helling 3D Laser Scanning Spray, Lot# 38580, Helling GmbH, Heidgraben, Germany, particle size. 2.8 µm). The specimen was placed and secured to a holder in the scanning platform by placing modelling clay in the buccal side of the appliance. This allowed the camera module to record the regions of interest, i.e., the palatal area and the extension. A successful scan comprised all regions of interest without any holes or non-recorded regions, as well as no major artefacts surrounding the scan. The sample was exported in a STL (Standard Tessellation Language) format. 

### 2.2. Automatization of the Measurement Protocol

CAD software, Rhino 7 and the related Grasshopper 3D (GH), were employed as tools to automate the measurement protocol. Grasshopper is a visual programming language and environment that operates within Rhino 7. This extension allows the use of the modelling tools within the CAD software, as well as the addition of custom-made scripts in different programming languages (C# script, VB script, Python script), in order to control or automate certain complex processes. This is achieved via an algorithmic diagram or workflow. Moreover, it allows the use of different GH open-source plugins from their plug-in community service (www.food4rhino.com, Robert McNeel & Associates, accessed on 20 November 2021). For this study, two plugins were employed:

- Human UI plugin (Version 0.8.1.3., year 2019, developed by A. Heumann, M. Syp, N. Holland, B. Ringley).
- Elefront (Version 5.0.0., year 2022, funded by Front Inc. and developed by Keyan Rahimzadeh, Ramon van der Heijden, and Alan Tai).

**Figure 2:**
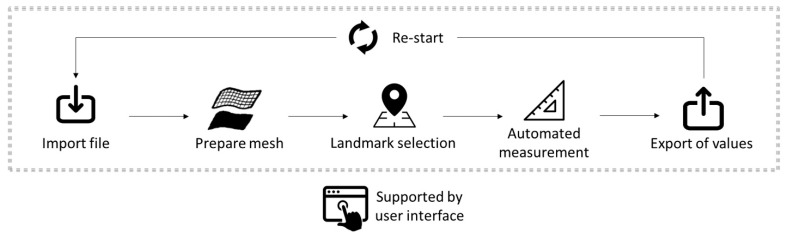
Proposed workflow for a standardized and automated device measurement. All the steps are supported by a user interface (UI).

The proposed solution for a standardized and automated measurement is shown in Figure 2. The process starts by selecting and importing an .stl format file of a TPP. The imported mesh file is then processed to allow for a smooth measurement of this part. Later, main orientation planes are created, on which the extension measurements are based. For this, three user-selected landmarks are placed in the base plate. From here, the program will automatically measure all desired dimensions. Later, the user is able to export this set of values, as well as re-start a plate measurement. In the following subsections, more detailed description of the programming clusters for the steps presented in Figure 2 is given.

#### 2.2.1. User Interface (UI) Creation and Editing Tools

The entire UI development process was carried out by the Human UI plugin. These commands were employed for every step within the workflow presented in Figure 2 and can be grouped into three main categories: main commands, UI tools and customization commands. 

**Figure 3:**
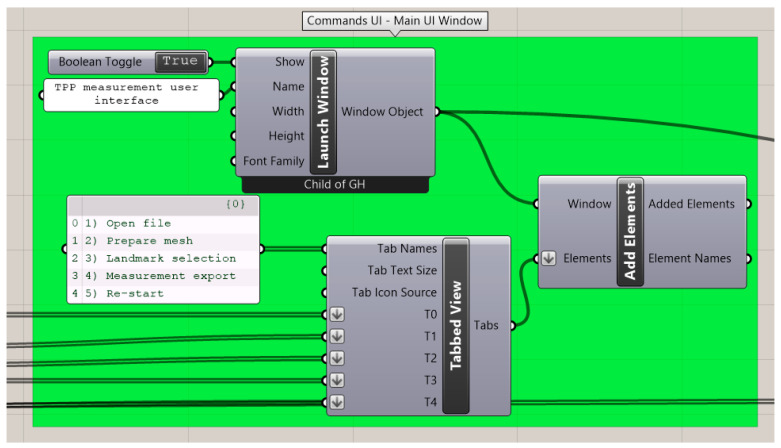
Main user interface (UI) commands from Human UI plugin for the generation of the main UI window and UI tool organization. Green background stands for UI commands.

**Figure 4:**
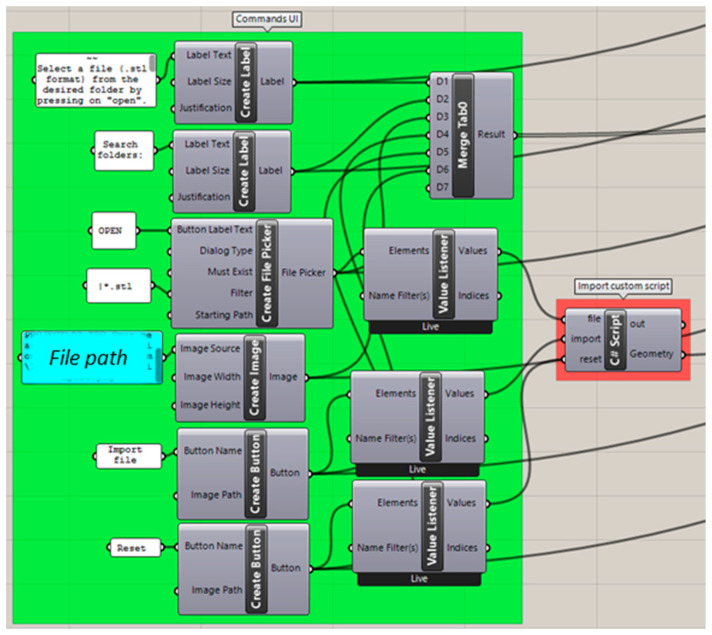
GH code section for selecting and opening the patient .stl file. Background colour coding: UI commands (green), custom script (red). Panels: customized text (white), file path (blue).

Main commands (Figure 3) used for this study were “launch window” for UI window creation, “add elements” for including all UI tools in the user interface and “tabbed view” for sectioning the different parts of the UI in a menu. UI tools are considered those that need to appear in the UI window for user manipulation or outsourcing information. These were added in the main window by “add elements” commands, in this case by organizing them by “tabbed view” (Figure 3). For tool organization, “create grid” and “create expander” commands were employed, whereas “create label” was employed for text addition. “Create button” was employed for cases where user interaction was needed to activate program command lines, as well as to control the process of the measurement process. Activation or non-activation of the button was obtained by a “value listener” command as a true or false value, which was the key component of activation for command parts (Figure 4).

Finally, the most important customization commands employed in this study were “element positioning” (for organization of elements in the UI), “element appearance” (colour, letter size etc. selection for user interface tools) and “custom preview” (colour, transparency and other options for displaying elements in the Rhino visualization window).

#### 2.2.2. Importing Files

As the first step in the workflow, an .stl file needs to be selected from a folder. For this, the command “Create File Picker” (HU) was employed, which allows for a button-activated opening of the Windows file explorer (Figure 4). The command was set so that the files of any user-selected folder will be filtered by the .stl format by default. Once selected, the related file path was used as input for a custom-made C# script command. This script is based on the activation of the Rhino app command window. The custom program allows for UI button activation of actions, such as importing and resetting of the part selection (Figure 4).

#### 2.2.3. Mesh Preparation

**Figure 5:**
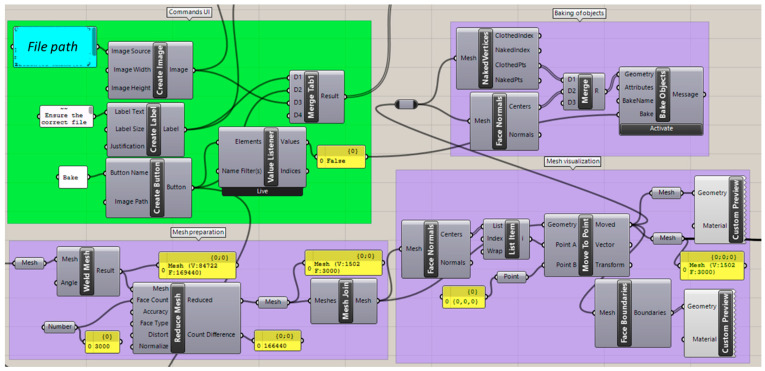
GH command for preparing and processing the mesh. Background colour coding: UI commands (green), conventional GH commands (purple). Panels: customized text (white), file path (blue), control panel (yellow).

The imported file was processed to facilitate user manipulation and execution of commands within the program (Figure 5), as well as reduce computing time. For starting, creases of the imported files are merged into a mesh by means of the “weld mesh” command (default angle = π).

**Figure 6:**
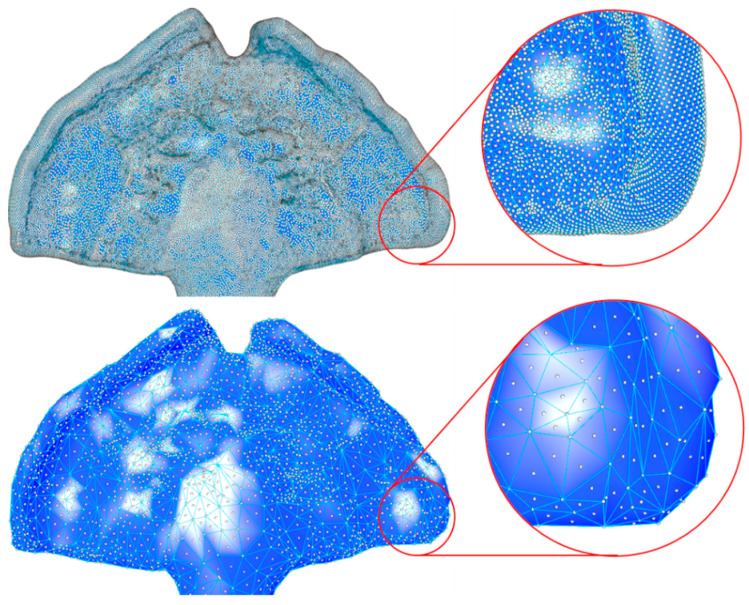
Example of the reduction of mesh face count. Before processing (**top**), the part consisted of 165,000 faces, and after processing (**bottom**), the value was decreased to 3000. The large number of face centres and vertices (baked Rhino objects as white points) makes selection of landmarks and the visualization of the part difficult.

Thereafter, the command “reduce mesh” was employed to reduce the face count to 3000. This step is necessary to reduce the extra artefacts from the scanner, as well as reduce the number of vertices available for the next landmark selection. The face count was set to maintain the quality and accuracy of the part as high as possible, while reducing the number of vertices, to enable the user to distinguish between these points (Figure 6). For safety, a “mesh join” command was inserted, so that the program does not recognize all as separate objects but as a single entity, in case the file consists of multiple parts (possibly scanning artefacts).

For improvement of the visualization of the object, the part was located in the middle of the visualization board by moving (“come to point” command) the first triangle centre point (“face normal” command) to the origin (0, 0, 0) of the coordinate system. Display colour, textures and reflection were defined for the face boundaries and the solid colour of the part by “custom preview”. As setup, a custom material was created to ensure a lack of transparency and provide a good contrast of the deep valleys and peaks within the palatal plate area (Figure 6), which was necessary for landmark selection.

Finally, the centres and vertices of the mesh triangles were baked (Figure 6). Baking is understood as the translation of a fictional visualization into a physical object in the main Rhino display interface, without which the manual manipulation, edition or selection of parts in the main Rhino program will not be possible. That is, the part only exists in the GH environment until baking allows for its translation to the main program.

#### 2.2.4. Landmark Selection

**Figure 7:**
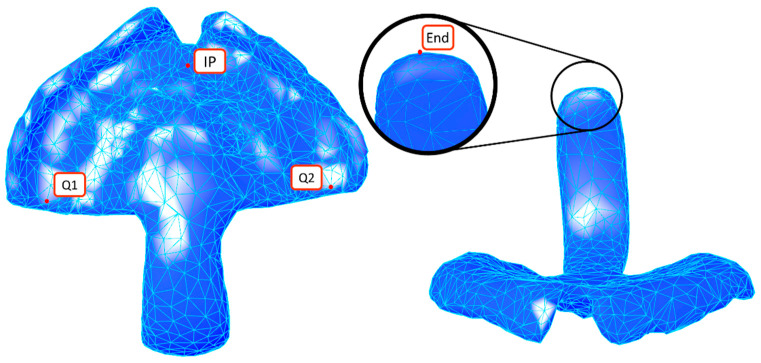
Landmark placement of incisive papilla (IP), end of the tuber maxilla region (Q1, Q2) and end of the extension (end).

As the face centres and vertices were baked beforehand, these objects can be manipulated in the main Rhino visualization window by the user. The main user interaction comes from the selection of the landmarks from where the complete measurement was planned. For that, four landmarks were defined (Figure 7): incisive papilla (IP), end of the tuber maxilla area (Q1, Q2) and the end part of the extension (end). These were selected as they are the most visible or recognizable for the user, and they are constant elements to be found in every fabricated appliance. The deepest areas of the palatal area were used for the selection of IP, Q1 and Q2, which enables distinguishing between the custom materials placed in the previous step. IP consisted of the most forward and deepest point, whereas Q1 and Q2 reflected the most backward and deepest points. The end point consisted of the deepest vertice point.

**Figure 8:**
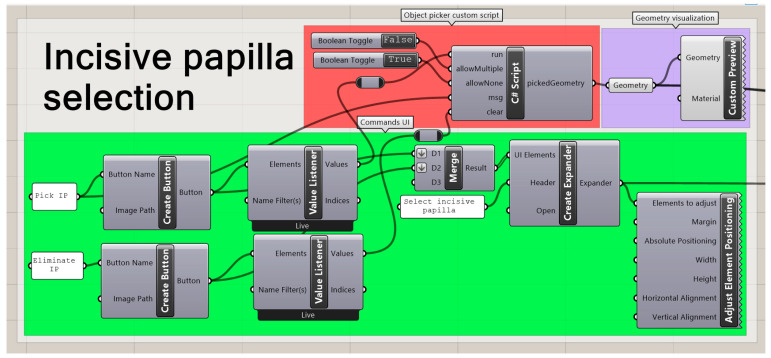
GH code for selection of a point within the measured part. Background colour coding: UI commands (green), conventional GH commands (purple), custom script (red).

In Figure 8, the code part for the selection of the IP is shown. The key command was created by a C# script, where a button activation allowed the selection or deselection of a point within the measured part to be carried out. The same command structure was employed for the remaining landmarks.

A control tool was included to verify that all landmarks were selected and, therefore, that the process could continue. If the geometries were not stored, a true value was obtained through the command “null item”. A child window of the main UI was created where the user can check for the completeness of the selection.

#### 2.2.5. Automated Measurement

**Figure 9:**
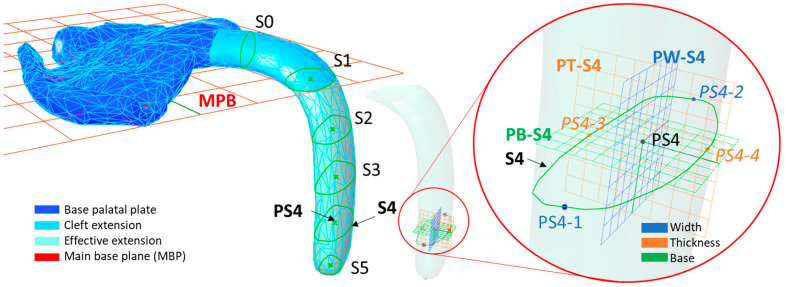
Isometric view of the measured part, with the sectional curves along the curve. S_0_ is the only section of the cleft extension, as this is the section with the lowest area. S_1_–S_5_ are distributed along the effective extension proportionally.

By employing the selected landmarks, a main base plane (MBP) was constructed. This was employed to divide the extension in between the “effective” and “cleft” extension (Figure 9), with the former being the main part responsible for the airway opening as it is in contact with the tongue base, whereas the latter is influenced by the shape of the cleft area, as well as employed dental technique. Different measurements were carried out to interpret the dimensions of the measured part. A summary of the employed construction and measurement names for the different sections, as well as their meanings, is shown in Table 1.

**Figure 10:**
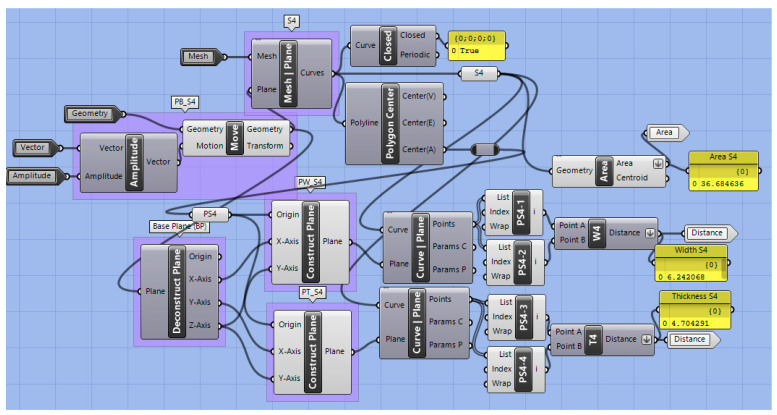
Clustered GH code part for sectioning and measuring the TPP extension. Example for Section 4.

Parallel planes to the base plane were constructed to create sectional planes within the extension (Figure 9). A total of five sectional planes were performed along the extension. Here, sectional curves (S1–S5) and their respective centroids (PS1–PS5; centre points of the sectional area) were calculated by the “curve and plane” component (Figure 10). From the centroid of the sectioning curve, two additional planes were created, which were perpendicular to the curve sectioning plane, as well as to each other (i.e., Section 4 in Figure 9; width plane PW-S4, thickness plane PT-S4;). From where the planes cut the sectional curves, intersection points were obtained (i.e., PS4-1 to PS4-4 for Section 4). In this way, the distance between the width (i.e., distance PS4-1 to PS4-2) and thickness (i.e., distance PS4-3 to PS4-4) of this sectional part could be obtained (Figure 9 and Figure 10). In addition, the area of the curve was obtained by the “area” command.

While the same procedure was used for all sections along the effective length of the extension, a different one was employed for the cleft side extension (Figure 9). This portion of the device is the one most influenced by the technician’s manufacturing, and, despite its shape, is less related to the opening of the airway; this section is comprised of the most extreme values of thickness and width. Thinner and narrower structures are often found to avoid pressure marks and aid physiological soft palate movements. Such values are interesting, as they are key information for the assessment of the quality of the device, as well as for preventing its breakage and respective complications. Therefore, an extra section (S0) was implemented in this region (Figure 9) following the same protocol. Instead of sectional planes parallel to the base plane, perpendicular ones were created along the cleft extension, dividing it in five equal segments. Areas of these sections were compared, selecting the smallest one as “Section 0” (S0) (Figure 9), or as the area where the most extreme values happened. Thickness and width for this area were also recorded by employing the procedure explained above (Figure 9 and Figure 10).

**Figure 11:**
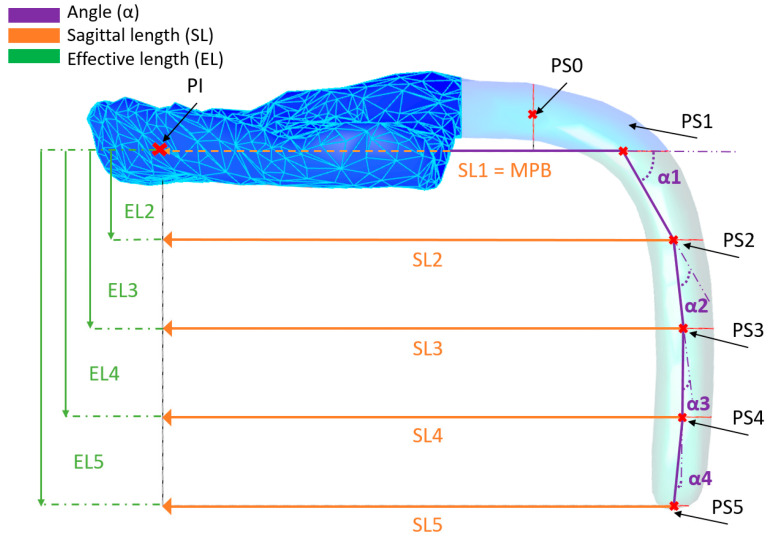
Lengths and angles of the extension. Sagittal (SL) and effective length (EL) information was given per section. SL1 had the same orientation as the main base plane (MPB). All sagittal length and effective lengths are parallel to each other. Moreover, angles (α) of the extension were calculated.

**Figure 12:**
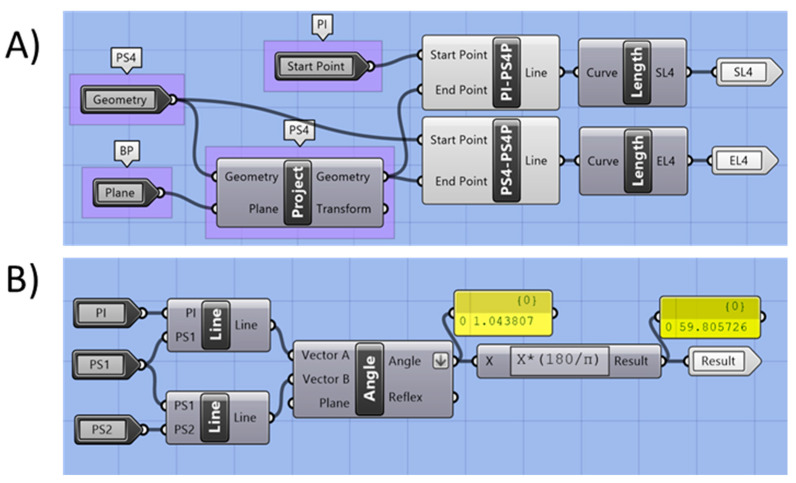
Clustered GH code part for calculation of lengths and angles. (**A**) Effective and sagittal lengths related to Section 4 (S_4_). (**B**) extension angle for the example α_1_.

Moreover, sagittal and effective lengths were obtained. These comprise distances of the sagittal and effective length directions for every single centroid (PS_0_–PS_5_) (Figure 11). For that, centroids were projected to the main base plane (Figure 12; i.e., PS_4_ projected to the main base plane becomes PS_4_-P). The distance between the centroids and their projection was recorded as the effective length, whereas the distance between PI and the projected point was recorded as sagittal length (Figure 11). 

For the extension inclination description, angles along the curve were calculated. Lines were created by connecting the centroids (command “line”, Figure 12), creating a skeleton of the extension (Figure 11). Angles in between lines were calculated in a clockwise direction. For the first main angle, the incisive papilla (IP) was connected to the first centroid (PS_1_), as this is where the major bending of the structure occurs (Figure 11). Moreover, effective extension volume was calculated for the effective extension segment (V_eff_), by the command “volume”.

#### 2.2.6. Exporting of Values

**Figure 13:**
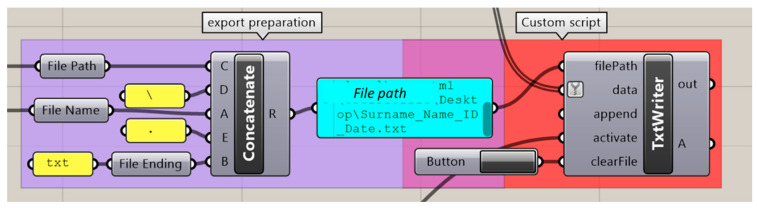
GH commands for measurement export. Background colour coding: Conventional GH commands (purple), custom script (red).

For exporting the measured values, the command was prepared so that values could be exported in a .csv format with a user-given name and a user-selected folder. The user name was obtained by a “text box” HU command, and a “file picker” was used to gather the folder location. This information was combined to the expected format (.csv) by the “concatenate” command. This information was followed by a VB custom script (Figure 13), which is responsible for the export action. This action was again tied to a user-activated UI button. As a control tool, a child window was placed to notify the user in case an export of an empty .csv file is attempted, which was identified by using “null item” when receiving the data in the VB script.

#### 2.2.7. Re-Starting of the Program

Once all the steps of the workflow are performed, the measurement program can be restarted. For that, a C# script was employed. This script was used to activate the Rhino interface command window, where the commands were given for selecting and eliminating all objects from the display interface. The script was setup to be activated by a UI activation button.

### 2.3. Case Study and Measurement Example 

Three different TPP case scenarios were employed to test the functioning of the proposed program. The three cases corresponded to different clinical pictures (Table 2). This was approved by the institutional ethics committee of the University Hospital Tübingen (approval number: 455/2019BO2) and was performed according to the Declaration of Helsinki in its current version. Case A was based on a newborn who received the first plate, which is expected to be small. Case B and C corresponded to two patients of similar age and size who received their second plate. The three cases were used to test the program for measuring slighter extension configurations as a means to compare cases that should be more similar theoretically.

## 3. Results

### 3.1. Performance of the Measuring Program and UI

**Figure 14:**
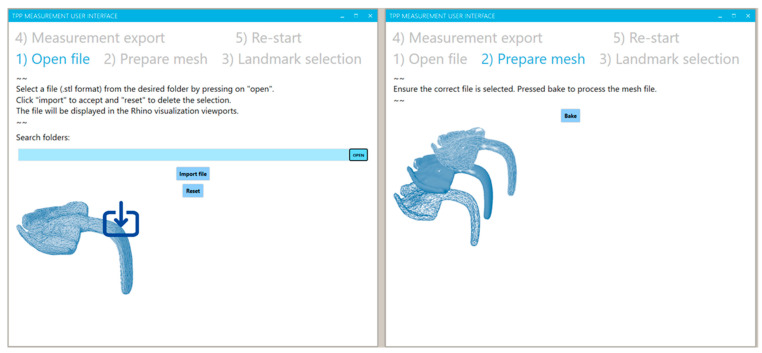
User interface view for the tabs of “open file” (**left**) and “prepare mesh” (**right**).

By means of the UI, the user was able to follow the workflow steps in order by clicking on the different tabs. By default, the first tab is always open when initializing the program. The UI provided explanatory text and pictures to guide the end user throughout the process. In the first step (Figure 14), the user was able to choose an .stl from any folder in the device by the “open file” button. The selection was confirmed by the button “import file”, and in case of importing a wrong file, the import could be eliminated by the “reset” button. Next, by activating the second tab (“prepare mesh”), the user needed to give permission for the mesh processing by a button activation (“bake”) (Figure 14). This permission allowed for the largest computer processing to be activated. The user needed to press it when the imported file was the suitable one, so that processing time could be saved in case of an incorrect selection, in contrast to processing directly and without user permission after the file import. 

**Figure 15:**
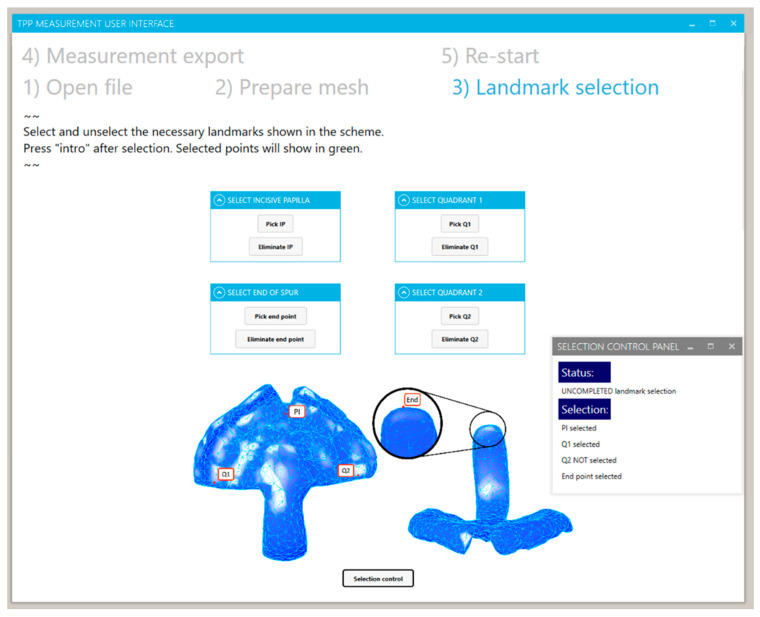
User interface view for the “landmark selection” and “selection control panel” for the completeness control of the process.

Later, the landmarks for the measurement basis and orientation were placed by the user. For that, the user was required to activate the landmark selection in the UI and subsequently select the appropriate vertices or point related to the landmark (Figure 15). The selection was carried out in the main Rhino visualization window, where the selected point would turn green. The selected point could be eliminated and the selection process carried out again by the button “eliminate”. Moreover, the user could activate a control window by the “selection control” button, which allowed a feedback about the completeness of the selection procedure and identifying which point selection was missing. This control step was important to establish, as the measurement could only be executed by selecting four landmarks. Once the selection was completed, all points, lines, planes, etc. needed for the measurement were automatically created.

**Figure 16:**
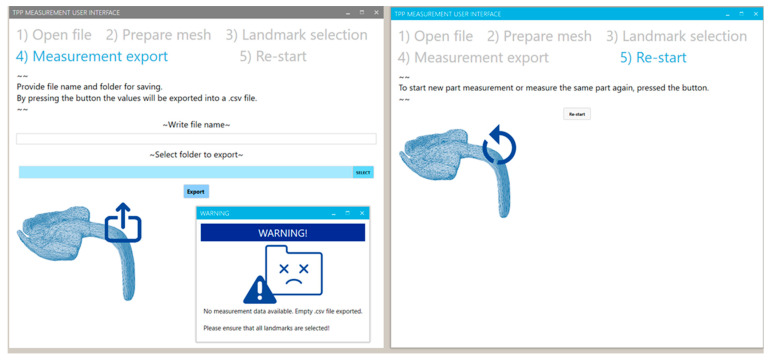
User interface view for the tabs of “measuring export” (**left**) and “re-start” (**right**). For an uncompleted measurement protocol or export document, a warning window appears (**right**).

In the tab “measurement export”, the user could define a file name and a folder for export. By selecting the “export” button, all measured values will be exported into a .csv document. In case any landmark has been eliminated or the measuring process has not been activated, the program will execute an error window (Figure 16) informing about the export of an empty document. Finally, the user could activate the button in the re-start tab (Figure 16), and all of the performed changes and processes can then be eliminated, setting the program to default mode. The user can re-start the process with a new measuring file in the first tab. For more detailed insight into the functioning of the proposed program, see the supplementary data.

### 3.2. Results of Case Study

A total of 32 values could be obtained from three different TPPs (Table 3). All values were based on the reference placed by the selected landmarks and were automatically exported. Diverse extension configurations were measurable, and differences in measurements results could be identified. 

**Figure 17:**
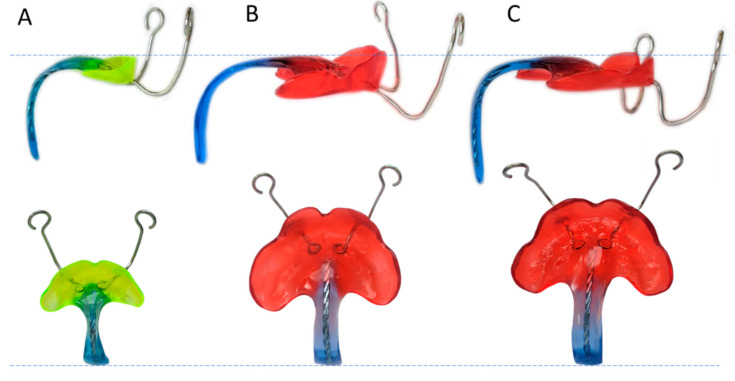
Three TPPs from different patient scenarios (Case A–C, see Table 2). Images are to scale and thus, show the real-life dimensional differences between the different TPPs.

Large dimensional differences were observed between case A and the other cases, for example for EL and SL (Table 3, Figure 17). Minimum width and thickness values were recorded in the cleft extension of case A, which were almost half the recorded values in B and C. Similar values for the first angle (α_1_) were obtained for all scenarios. For cases B and C, differences in effective length and sagittal length were already perceptible visually (Figure 17), but a quantification of the difference between plates could be obtained through the program. For example, the EL_5_ differed by 10 mm, whereas the SL_5_ differed by 8 mm. In this way, case C had a more frontally positioned and longer velopharyngeal TPP extension than case B.

## 4. Discussion

In this study, a methodology to automatize measurements of personalized medical appliances with anatomic shapes is proposed. In particular, the study provides a solution for measuring orthodontic appliances known as the TPP, where parameters such as length, width and angle could help to standardize and improve treatment in the future. 

While the shape of the TPPs could not be evaluated and measured manually with common methods, such as callipers, the proposed program proved to be useful for this goal. It was possible to measure and compare real TPPs, where small differences in the velopharyngeal extension could be quantified. A complete picture of the morphology of the extension was obtained, independent of its configuration. 

Given the nature of the production of the TPP, as well as the proposed methodology, it has the same problems with landmark extraction and measurements as seen in medical imaging techniques [6]. For example, the difficulty in quantifying the accuracy of the landmark selection and its measurement is also related to the employed rater. This is because fully automated systems for 3D model measurements cannot yet be obtained in medical imaging. Individual variations in shape and distortions from the normal shape, represented by malformations or other issues, render complete automation difficult [6].

While medical disciplines still rely on the identification of landmarks for measurement purposes, new technologies have the potential to enhance this process by reducing employed time and improving the reliability of the measurements [6,10]. In this way, clinically relevant distance and angle measurements, as well as shape analysis and comparison, can be simplified. In the current study, the three landmarks from the base plane affect the orientation of the plane. Nonetheless, the effect is expected to be low. Even with a selection deviation of 0.5–1 mm in the landmark, the plane’s orientation would not change significantly enough to cause a major disruption in the sectioning of the extension and the respective measurements. The largest effect is expected on the calculation of the sagittal length, as a more forward selection of the papilla will result in a longer sagittal length. When compared to any conventional measurement method, such as the use of a calliper, selecting the same exact point for a landmark for every single measurement is done only once. This means that the landmark does not need to be reselected for every measurement. Conventionally, it will need to be re-selected, adding a new random error to the measurement, whereas with an automated program, the landmark stays constant across all measurements. As a benefit to the operator, this reduces the occurrence of errors and improves other aspects, such as measurement duration.

When it comes to software selection, the combination of Rhinoceros and Grasshopper proved to be a user-friendly solution through the help of its visual programming environment. Despite the user needing a basic knowledge of CAD measurement processes and their related workflow, an automation process, as well as a user interface, can be created with comparatively little investment and time. The process is further simplified thanks to the UI, as it allows further use of the program for clinical staff without CAD or programming knowledge. This can be a major advantage when implementing measurement automation for smaller projects or research niches, such as in the current study, in both medicine and other fields [11]. The development of more sophisticated self-developed programs for measuring, with an improved user interface or executable programs, as well as the inclusion of techniques such as machine learning and artificial intelligence, is not applicable for such small projects. Increased time, investment and human resources cannot be easily justified for research performed for diseases with low prevalence, as it has only low commercial potential. 

Regarding the clinical relevance, the proposed measurement methodology can be used to gather information on successfully used plates and their relationship to the clinical picture. This could then be employed for the creation of guidelines to further standardize TPP production for different patients, which could potentially reduce the fitting time or endoscopic procedures, as well as reduce the workload in the orthodontic dental laboratory. Moreover, it would provide inexperienced centres interested in adopting the TPP treatment with some orientation for treating their patients that could facilitate its implementation. Eventually, the developed program could act as a bridge for future design and production of TPPs, as well as improvement of the therapy. Additionally, this kind of methodology could successfully be applied in other fields of personalized devices, as the employed software proved to be easy to develop and the commands offer a variety of options for automated measurement. Its successful adaptation in the remaining applications is mostly dependent on the possibility for landmark or specific structure detection for the referencing of the part.

To mention some limitations of this study, a completely autonomous measuring procedure was not possible to obtain, as some level of user interaction is necessary and, therefore, a user influence on the obtained values cannot be avoided. Regardless of the few landmarks employed, while minimal, an error in the recorded values can be expected. Optimization of the program can be expected after measuring a larger set of TPPs, in order to address and measure a wider range of possible TPP modifications and scenarios. Moreover, a successful scan meeting all of the mentioned requirements is a necessity, where all of the identifiable landmarks and areas to be measured can be identified. Artefacts can make the processing and further measurement of the sample more difficult, as well as increase the chances of program crash.

## 5. Conclusions

In summary, this work focused on proposing a methodology that exploits the potential of CAD technologies for automating the measurement of individualized devices with complex anatomical shapes. A custom-program based on Rhinoceros 7 and Grasshopper was presented to measure an orthodontic appliance employed for Robin sequence treatment, known as the Tübingen Palatal Plate (TPP). The program proved to be useful for the measurement and comparison of real TPPs, where small differences in the velopharyngeal extension could be quantified successfully. In this way, measurement time and human error were reduced, while offering a wide range of measurements describing the shape of the extension of the appliance. Further studies need to evaluate the relationship between the device’s physical dimensions and clinical parameters in order to further optimize this treatment, as well as establish a guideline that serves to shorten the fitting of the TPP for different clinical pictures. This measuring methodology could ultimately be used for the completeness of clinical records and the improvement of the therapy.

## Figures and Tables

**Table 1:** Resume of recorded values and their respective acronyms. Example for Section 4 (S_4_).

Construction Names		Measurement Names	
Acronym	Meaning	Acronym	Meaning
S_4_	Curve Section 4	A_4_	Area of S_4_ (mm^2^)
PB_4_	Base plane S_4_	W_4_	Width S_4_ (mm)
PS_4_	Centroid point S_4_	T_4_	Thickness S_4_ (mm)
PW-S_4_	Plane for width S4	EL_4_	Effective length to S_4_. Distance PI to PS4 (mm)
PT-T_4_	Plane for thickness S4	SL_4_	Sagittal length to S_4._ Distance PI to PS4 (mm)
PS_4_-P	Projection of PS4 on main base plane	α_4_	Angle (°)

**Table 2:** Clinical picture of three different patients (case A–C) receiving TPP treatment.

Case	Gender	Age (Days)	Gestational Age		Head Circumference (cm)	Plate Number
			Weeks	Days		
Case A	Female	18	37	6	34	1
Case B	Male	162	40	2	44	2
Case C	Male	165	38	1	43	2

**Table 3:** Measured values in the proposed TPPs from three case scenarios. A total of 32 values per part were automatically obtained from the program. S: Section, EL: Effective length, SL: Sagittal length, A: Sectional area, W: Width, T: Thickness, Veff: Effective volume, α: angle.

Values		Case A	Case B	Case C	Values		Case A	Case B	Case C
**S_0_ (mm)**	EL_0_	1.05	5.14	3.61	**S_3_ (mm)**	EL_3_	10.57	9.32	13.29
	SL_0_	23.21	34.01	27.79		SL_3_	28.63	45.59	37.83
	A_0_	7.17	17.04	15.18		A_3_	19.67	28.28	22.19
	W_0_	3.38	7.93	6.10		W_3_	8.00	5.25	2.97
	T_0_	2.53	2.50	2.74		T_3_	2.79	3.31	4.14
**S_1_ (mm)**	EL_1_	0.00	0.00	0.00	**S_4_ (mm)**	EL_4_	15.86	13.98	19.93
	SL_1_	24.58	42.08	34.55		SL_4_	29.00	45.97	37.42
	A_1_	14.01	26.16	20.95		A_4_	21.45	24.04	24.07
	W_1_	4.77	7.94	6.97		W_4_	4.17	3.83	3.99
	T_1_	3.51	3.52	4.73		T_4_	3.59	3.99	4.36
**S_2_ (mm)**	EL_2_	5.29	4.66	6.64	**S_5_ (mm)**	EL_5_	21.14	18.63	26.58
	SL_2_	27.43	44.54	37.39		SL_5_	29.12	45.91	35.83
	A_2_	16.74	29.25	18.72	**Angles (°)**	α_1_	61.67	62.27	66.91
	W_2_	6.64	5.66	2.69		α_2_	8.78	8.17	7.44
	T_2_	2.85	3.56	3.81		α_3_	6.11	10.4	4.45
**V_eff_ (mm^3^)**		374.20	475.39	547.17		α_4_	15.67	15.13	19.4

## Data Availability

Data available from the corresponding author upon reasonable request.

## Supplementary Materials

The following supporting information can be downloaded at: https://www.mdpi.com/article/10.3390/bioengineering9120773/s1, Video S1: TPP_Rhino_video.mp4.
